# The impact of using a 4K 3D surgical microscope during associated liver partition and portal vein ligation for hepatocellular carcinoma treatment: A case report with operative video

**DOI:** 10.1016/j.ijscr.2021.106195

**Published:** 2021-07-15

**Authors:** Kenta Doden, Masahiko Kawaguchi, Takahiro Yoshimura, Yoshitaka Iwaki, Hideaki Kato, Toru Watanabe

**Affiliations:** Department of Surgery, Yokohama Sakae Kyosai Hospital, 132 Katsura-cho, Yokohama 247-8581, Japan

**Keywords:** Hepatocellular carcinoma, Minimally invasive, Video microscopy

## Abstract

**Introduction:**

Associated liver partition and portal vein ligation for staged hepatectomy (ALPPS) is complicated by bile leakage or liver failure, especially in patients with hepatocellular carcinoma (HCC). Precise surgical performance supported by high quality intraoperative surgical visualization is essential to prevent mortality. Therefore, we aimed to investigate, for the first time, the effects of introducing a surgical microscope (ORBEYE™) intraoperatively during a stage I ALPPS.

**Presentation of case:**

The patient was a 77-year-old male patient with a 9-cm right hepatic lobe HCC. 4K-3D surgical microscope-assisted ALPPS was performed to manage the insufficient future liver remnant following right lobectomy. Hilar dissection was performed first; thereafter, the right portal vein was ligated, and the right hepatic artery and right hepatic vein were encircled by surgical tape. The parenchyma was split along the ischemic demarcation line with indocyanine green (ICG) fluorescence navigation using the microscope. The remnant liver volume and function increased without postoperative complications.

**Discussion:**

Laparoscopic approach for ALPPS benefits from enhanced intraoperative visualization in a deep, narrow operative field. However, a laparoscopic procedure requires an experienced learning curve and a longer operation time, whereas using the 4 K 3D digital microscope requires no technical demand. Secondly, it provided an excellent operative view during ALPPS.

**Conclusions:**

To our knowledge, this is the first report on the intraoperative application of the ORBEYE™ surgical microscope in hepatic surgery with 4K3D imaging and ICG-fluorescence navigation, which minimized the invasiveness of ALPPS and ensured high safety and precision.

## Highlights

• ALPPS enhances future liver remnant hypertrophy.
• ALPPS enables tumor resection 4–6 weeks earlier than portal vein embolization.
• Precision in surgery requires high-quality intraoperative visualization.
• Accurate visualization is needed to prevent mortality after ALPPS in HCC patients.
• We demonstrate ALPPS with 4K3D microscopy system with ICG-fluorescence navigation.

## 1 Introduction

Liver malignancies are usually best treated by anatomical resections to ensure long-term survival [1], [2]; to assure this, surgeons aim at performing R0 resections even when patients have locally advanced hepatic tumors. However, R0 resections may leave an insufficient future liver remnant, which can develop post-hepatectomy liver failure (PHLF) [3]. Although insufficient future liver remnant has been conventionally treated by portal vein embolization (PVE) [4] to increase the future liver volume and function, it takes over 4–8 weeks to gain adequate future liver remnant hypertrophy while the tumor growth may continue.

Associated liver partition and portal vein ligation for staged hepatectomy (ALPPS) emerged in 2012 [5] as an advanced surgical technique that enhances future liver remnant hypertrophy. The technique has demonstrated the ability to resect tumors 4–6 weeks earlier than PVE [6]. However, ALPPS is complicated by bile leakage and PHLF. Moreover, in previous reports on patients with hepatocellular carcinoma (HCC), significantly less future liver remnant growth and ≥90-day mortality were achieved after performing stage I ALPPS [7]. Therefore, surgical success of the stage I procedure is essential to prevent mortality in HCC patients.

A newly developed 4K-3D video microscopy system, which has been introduced as an alternative to the surgical loupe or conventional microscope in the microsurgery field, is able to improve surgical performance by providing excellent image quality and reducing physical discomfort [8], [9]. Recently, it has been introduced to general surgery field [10], considering its superior ergonomic, imaging, and educational aspects. However, the application of 4K-3D video microscope systems in hepatectomies and/or ALPPS has not been presented yet. Therefore, we investigated the effectiveness of performing stage I ALPPS with intraoperative surgical visualization using a digital video microscope to maximize the technical safety and surgical performance. This work is reported by following the surgical case report (SCARE) guidelines [11].

## 2 Presentation of case

**Fig. 1:**
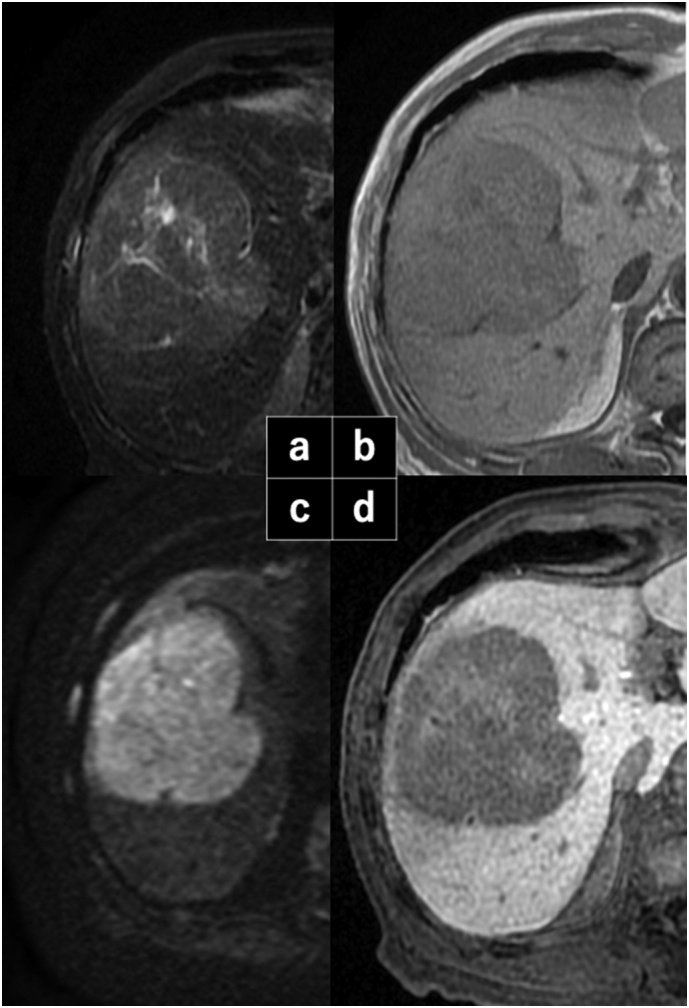
Hepatobiliary phase of gadolinium ethoxybenzyl-diethylenetriaminepentaacetic acid-enhanced magnetic resonance imaging. A 9-cm sized hepatocellular carcinoma in the right lobe of the liver was detected.

A 77-year-old man with a medical history of diabetes, arteriosclerosis obliterans, and alcohol consumption (three bottles of beer per day) was referred to our institution for a hepatic tumor by family physician. Laboratory evaluation detected abnormally high serum protein induced by vitamin K absence-II (PIVKA-II: 52200 MAU/ml), while other tumor markers, including alpha-fetoprotein (8 ng/ml), had no abnormalities. Hepatitis B surface antigen and hepatitis C antibody levels were normal. Computed tomography and gadoxetic acid-enhanced magnetic resonance imaging detected a 9-cm sized HCC in the right hepatic lobe [Fig. 1]. Although the patient had a Child-Pugh Score of 5, his ICG-R15 was 18%, which is insufficient for right hepatectomy according to Makuuchi's criteria [12]. The future liver remnant volume was 320 ml, whose function was evaluated with an estimated galactosyl human serum albumin (GSA) index [13] using technetium-99 m diethylenetriamine-penta-acetic acid-GSA (99mTc-GSA) scintigraphy. His left GSA index was 0.36, which was below the cut-off value of 0.38 for predicting PHLF [14]. Therefore, we planned to perform ALPPS for the right lobectomy (see Video 1).

**Fig. 2:**
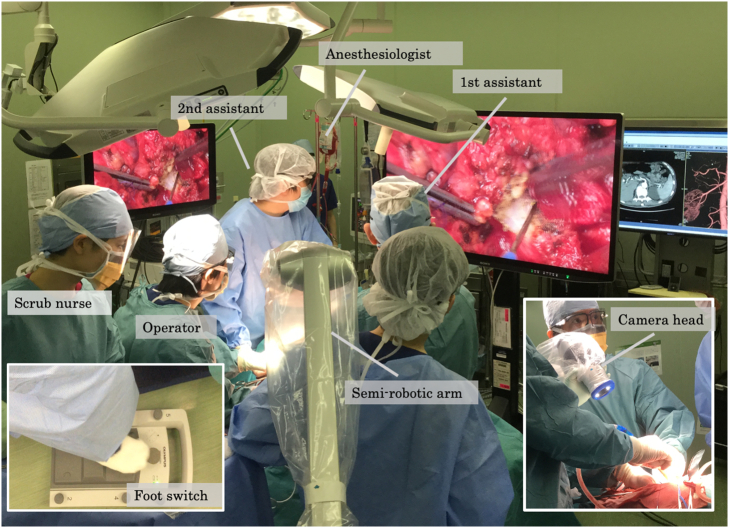
The intraoperative setup of the surgical microscope system (ORBEYE™). The surgical microscope was placed over the operative field with a 55-inch 4K3D monitor in front of the surgical team. The large 55-inch monitor enabled the operating team to view the 4K3D images simultaneously.

**Fig. 3:**
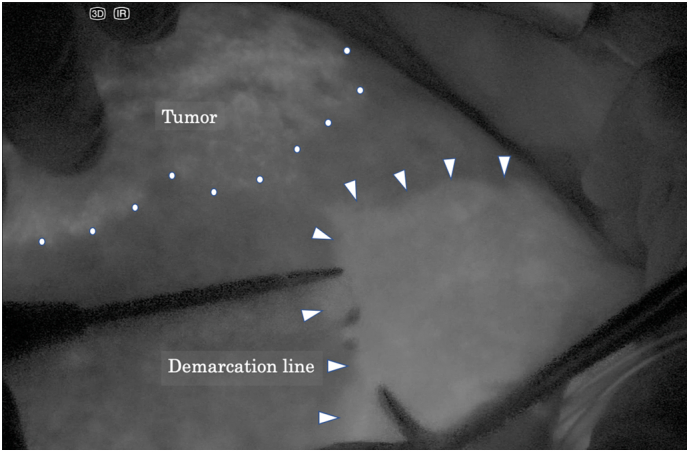
Indocyanine green fluorescence imaging-guided lobectomy using the surgical microscope system. The dotted line indicates the margin of the hepatocellular carcinoma. The arrows point to the ischemic demarcation line of the right lobe.

**Fig. 4:**
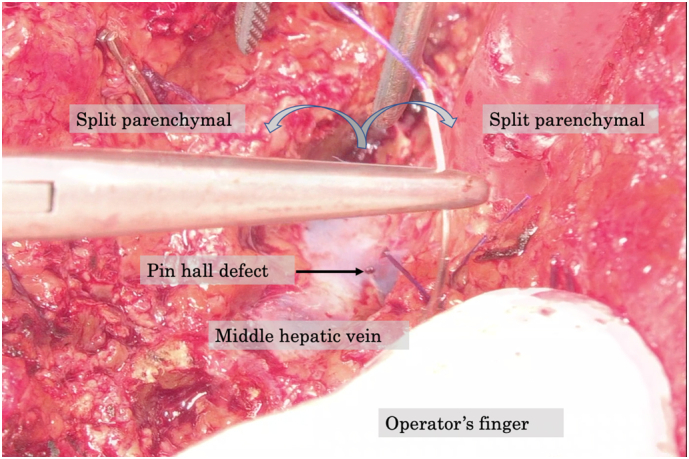
The exposed middle hepatic vein with microscopic imaging. The pinhole bleeding from the vein was repaired.

With the patient in a left hemi-lateral position, we performed a J-shaped incision. The surgical microscope (ORBEYE™, Olympus Co., Ltd., Tokyo, Japan) was set over the operative field and a 55-inch 4K3D monitor was placed in front of the whole operating system [Fig. 2]. After mobilizing the right hepatic lobe, the right hepatic vein was wound with surgical tape. Indocyanine green (ICG) dye was injected preoperatively, which enabled tumor identification by fluorescence imaging [Fig. 3]. Then, we separated the right hepatic artery and the right portal vein and applied non-absorbable sutures on both these vessels. The right portal vein was then ligated, but not divided; the right hepatic artery was temporarily clamped, and 2.5 mg of ICG was administrated via the peripheral vein. With ICG-fluorescence imaging navigation, we identified the ischemic demarcation line 40 s after the ICG injection [Fig. 3]. By ensuring no tumor exposure, which was enhanced, we performed the partial parenchymal split until the surface of the middle hepatic vein was exposed [Fig. 4]. The stage I operation lasted 285 min, and there was 150 ml intraoperative bleeding.

Future liver remnant volume increased from 320 ml to 502 ml; the left GSA index improved from 0.36 to 0.57 on postoperative day (POD) 14. This indicates the shift in hepatic function from the right hepatic lobe to the left hepatic lobe, as well as enlargement of the left hepatic lobe [14], [15]. Subsequently, a stage II operation was performed on POD 17 without any postoperative complications. The pathological specimen showed moderately differentiated HCC with a negative tumor margin.

## 3 Discussion

We report from our experience with this patient that 4K3D-microscopy and ICG-fluorescence navigation enabled a safe, precise ALPPS stage I procedure. Although ALPPS can be performed by open, laparoscopic, and robotic procedures, only a limited number of reports exist, with a considerable level of selection biases when comparing those approaches. When compared with the conventional open hepatic lobectomy, laparoscopic hepatic lobectomy showed perioperative advantages, including fewer complications and shorter hospitalization [16] because of the enhanced intraoperative visualization in a deep, narrow operative field, such as for mobilization of the right lobe. As laparoscopic hepatic lobectomy requires an experienced learning curve and a longer operation time, we preferred to perform an open 4K3D-imaging assisted hepatectomy with ORBEYE™, which provided excellent visualization, thus overcoming the challenges of laparoscopy. There is no skill required to use this surgical microscope and we need neither certification nor training. Surgeons could perform this video microscope assisted surgery as an extension of open surgery. Neither a certification nor training for ORBEYE™ **is** needed. This device enabled quick magnification change from 1× to 26× and 4K3D imaging, which improved the surgical performance [17]. This excellent camera view was stabilized by the flexible semi-robotic arm and easily controlled through the foot pedal. Moreover, the large 55-inch monitor enabled the entire operating team, including the anesthesiologist, nurses, and residents, to simultaneously view the 4K3D images [Fig. 2], which improved the surgical efficiency and education [8]. In our case, all surgical team members could observe the pinhole defect of the middle hepatic vein; hence, we repaired it immediately [Fig. 4].

Another advantage of this 4K3D surgical microscope is that it equips the ICG-fluorescence imaging system for ALPPS, which enables the highly sensitive identification of cancers [18] and specific liver segments [19]. HCC fluoresces because it retains the preoperatively injected ICG from the liver function test because of biliary excretion disorders in the cancerous tissues. As the interval required between the ICG injection and operation ranged from 1 to 7 days in patients with HCC, we performed the ICG test 4 days before surgery and obtained sufficient fluorescence of the tumor. For identifying liver segments, ICG should be administered after clamping vessels connected to the segment to obtain better signal-to-background contrast. A recent study demonstrated lower blood transfusion and postoperative complication rates, and higher negative margin rates in patients undergoing ICG-fluorescence guided right hemi-hepatectomy [19]. In our case, the tumor was not exposed, and no postoperative bile leakage occurred.

While ORBEYE™ has invented for reducing operator's workload in microsurgery, it has reported that residents who participated in neurosurgery with the microscope judged that eyestrain was strong [20]. For operative members who doesn't get used to viewing 3D imaging, the glasses which convert 3D images to 2D images are available. The high initial cost of this surgical microscope ($412,000, including the camera, scope holder, and 4K3D monitors) can limit its applicability. However, considering its multiple advantages, the system and its costs can be shared with other departments, e.g., neurosurgery and plastic surgery. In addition, once the microscope system is introduced, just a drape for the semi-robotic arm will be an operating cost.

Because only one surgical case was presented without a learning curve, this was a major limitation of this case report. More application experience with this surgical microscope would contribute to a noticeable improvement in surgical results, especially in the operation time.

## 4 Conclusions

To our knowledge, this is the first report on using a surgical microscope in hepatectomy, demonstrating that the ORBEYE™ 4K3D microscopy system can provide 4K3D images and ICG-fluorescence navigation. The application of this surgical microscopy system can enhance the safety and precision of surgery and minimize the invasiveness of ALPPS.

The following are the supplementary data related to this article.

Video 1 4K 3D surgical microscope - assisted ALPPS for hepatocellular carcinoma Video 1

## Abbreviations

ALPPS: associated liver partition and portal vein ligation for staged hepatectomy
HCC: hepatocellular carcinoma
ICG: indocyanine green
PHLF: post-hepatectomy liver failure
PIVKA-II: protein induced by vitamin K absence-II
POD: postoperative day
PVE: portal vein embolization
99mTc-GSA: technetium-99m diethylenetriamine-penta-acetic acid-galactosyl human serum albumin

## Ethical approval

The need for ethical approval for this report was waived by the Ethics Committee of the by our institution.

## Funding

No grant support or funding was received for this case report.

## CRediT authorship contribution statement

K.D. prepared the original draft. M.K. reviewed and edited this report. M.K., T.W., and H.K. performed the operation. T.Y. and Y.I. contributed to perioperative management and data collection. All authors have read and agreed to the published version of the manuscript.

## Guarantor

Kenta Doden, corresponding author of this article

## Research registration number

Not applicable.

## Consent

The subject gave informed consent, and patient anonymity was preserved. A copy of the written consent is available for review by the Editor-in-Chief of this journal on request.

## Availability of data and materials

The datasets supporting the conclusions of this article are included within the article.

## Declaration of competing interest

The authors have no competing interests to disclose.
